# Indications of the extraction of symptomatic impacted third molars. A systematic review

**DOI:** 10.4317/jced.56887

**Published:** 2021-03-01

**Authors:** María Peñarrocha-Diago, Octavi Camps-Font, Alba Sánchez-Torres, Rui Figueiredo, María-Angeles Sánchez-Garcés, Cosme Gay-Escoda

**Affiliations:** 1 DDS, MS, PhD. Assistant Professor of Oral Surgery. University of Valencia Medical and Dental School. Valencia, Spain. Researcher of the “Dental and Maxillofacial Diseases and Therapeutics” group of the Bellvitge Biomedical Research Institute (IDIBELL); 2 DDS, MS. Associate Professor of Oral Surgery. University of Barcelona Dental School. Barcelona, Spain. Researcher of the “Dental and Maxillofacial Diseases and Therapeutics” group of the Bellvitge Biomedical Research Institute (IDIBELL); 3 DDS, MS, PhD. Associate Professor of Oral Surgery. Coordinator of the Master of Oral Surgery and Buccofacial Implantology. University of Barcelona. Barcelona, Spain. Researcher of the “Dental and Maxillofacial Diseases and Therapeutics” group of the Bellvitge Biomedical Research Institute (IDIBELL); 4 MD, DDS, MS, PhD, EBOS. Associate Professor of Oral Surgery. University of Barcelona Dental School. Barcelona, Spain. Subdirector of the Master of Oral Surgery and Buccofacial Implantology of the EFHRE International University / FUCSO. Researcher of the “Dental and Maxillofacial Diseases and Therapeutics” group of the Bellvitge Biomedical Research Institute (IDIBELL); 5 MD, DDS, MS, PhD, EBOS, OMFS. Chairman of Oral and Maxillofacial Surgery. University of Barcelona Dental School. Barcelona, Spain. Director of the Master of Oral Surgery and Buccofacial Implantology of the EFHRE International University / FUCSO. Coordinator / Researcher of the “Dental and Maxillofacial Diseases and Therapeutics” group of the Bellvitge Biomedical Research Institute (IDIBELL)

## Abstract

**Background:**

A literature review was made to determine when third molar (3M) extraction is recommended in symptomatic patients and when it is not recommended.

**Material and Methods:**

A Medline (PubMed) and EMBASE search was made for articles related to indications for the extraction of 3Ms, published in the last 10 years and up until September 2018.

**Results:**

The electronic search yielded 175 articles. After eliminating duplicates, a total of 173 articles were subjected to review of the title and abstract. Only 19 studies were finally included in the systematic review. There was a well documented increase in morbidity associated to impacted 3Ms (non-restorable caries, fracture, infection, periodontal disease, repeated pericoronitis, cysts and tumors), and in the presence of disease, extraction was considered to be indicated. The extraction of 3Ms with signs and/or symptoms of periodontal disease improved periodontal health at the distal surface of the second molar. Postoperative quality of life of patients with symptomatic 3Ms and with disease improved after surgical extraction.

**Conclusions:**

Extraction is indicated in the presence of disease associated to an impacted 3M, whether symptomatic or not. In contrast, extraction is not indicated in the absence of infection or other associated disease conditions.

** Key words:**Third molar, periodontal disease, periodontitis, pericoronitis, dental caries, occlusal caries, mandibular cysts, osteomyelitis, odontogenic tumor.

## Introduction

One of the most important scenarios in dental practice, and particularly in oral surgery, is the presence of diseases and/or complications associated to wisdom teeth or third molars (3Ms), derived from eruption disorders that adversely affect the periodontal health of the neighboring teeth (1,2). Indeed, third molar extraction is the most frequent type of surgery performed by dental surgeons (3). Extraction and the indication of extraction must be based on scientific evidence allowing us to make solid decisions for the benefit of our patients (4). However, there is controversy regarding the prophylactic removal of asymptomatic impacted 3Ms without associated disease (5,6). In this context, it must be taken into account that “asymptomatic” does not discard the possible existence of disease (3,7).

An increasingly relevant concept in prophylaxis is being able to distinguish between patients with no molar symptoms but with associated disease and those with molar symptoms but no associated disease. In the presence of signs or symptoms produced by a 3M (pain, infection, local and/or regional swelling, etc.), patients tend to visit in search of the best possible treatment, which in most cases will consist of surgical or nonsurgical extraction, conditioned to cost-benefit criteria.

However, in many cases there are no such signs or symptoms, despite the presence of disease associated to the position of the third molar (periodontal pockets at the distal surface of the adjacent second molar, impacted molar follicle enlargement, cysts, root reabsorption, etc.) (8-11).

Thus, prophylactic extraction (i.e., removal of the tooth in the absence of symptoms and without disease) must be decided based on a number of prior considerations, of which two are particularly important: (a) What are the chances that the impacted 3M will cause disease at some point in the life of the patient? (b) What morbidity can be expected from removing the molar in a young patient under 25 years of age? This latter issue is clearly pertinent, considering the increasing life expectancy of the population (6).

The present literature review was made to determine those cases in which 3M extraction is recommended in symptomatic patients and in which cases it is not recommended.

In addition, we aimed to establish the indication for the removal of asymptomatic impacted 3Ms with or without associated disease, and to determine which patients with associated disease are likely to have a better outcome in terms of the appearance of complications.

## Material and Methods

The literature review was carried out based on the PRISMA criteria (12), and our search strategy was guided by the following modified PICO (Population, Intervention, Comparison, Outcome) question (13): What are the indications for extracting third molars that are impacted, produce symptoms or present associated disease?

-Electronic search

A Medline (PubMed) and EMBASE search was made for articles related to indications for the extraction of 3Ms, published in the last 10 years and up until September 2018. We used MeSH (Medical Subject Headings) terms as well as non-MeSH or Free-Text terms, combined with the boolean operators OR / AND as follows:

-MEDLINE (PUBMED):

((((“Molar, Third”[Mesh] OR wisdom teeth OR wisdom tooth OR third molar OR third molars) AND (“Tooth Extraction”[Mesh] OR removal OR nonextraction OR management))) AND ((symptomatic OR second molar OR periodontal health OR periodontal status OR probing depth OR periodontal pocket OR “Pericoronitis”[Mesh] OR pericoronitis OR impacted OR included OR occlusal caries OR cervical caries OR odontogenic cyst OR “Jaw Cysts”[Mesh]) OR “Osteomyelitis”[Mesh])) AND (indication OR indications)

-EMBASE:

(((‘molar tooth’/exp OR ‘molar tooth’ OR wisdom) AND (‘tooth’/exp OR tooth) OR wisdom) AND (‘teeth’/exp OR teeth)) AND (((‘tooth extraction’ OR tooth) AND removal OR tooth) AND (non AND extraction OR management)) AND (((((((((impacted AND third AND molar OR symptomatic) AND third AND molar OR second) AND molar OR ‘periodontal disease’ OR periodontal) AND health OR periodontal) AND status OR ‘probing depth’ OR ‘periodontal pocket’ OR periodontal) AND pocket OR ‘gingiva disease’ OR pericoronitis OR occlusal) AND caries OR cervical) AND caries OR ‘odontogenic tumor’ OR ‘jaw cyst’ OR jaw) AND cyst OR ‘osteomyelitis’)

-Inclusion criteria:

- Randomized clinical trials (RCTs) or non-randomized trials, observational cohort studies, case-control studies and case series (at least 10 cases) involving a cross-sectional design.

- Evaluation of the prophylactic extraction of impacted or non-impacted symptomatic third molars with associated disease.

-Exclusion criteria:

- Publications in languages other than English, French or Spanish.

- Preclinical / *in vitro* studies, finite element studies or necropsy studies.

## Results

**Figure 1:**
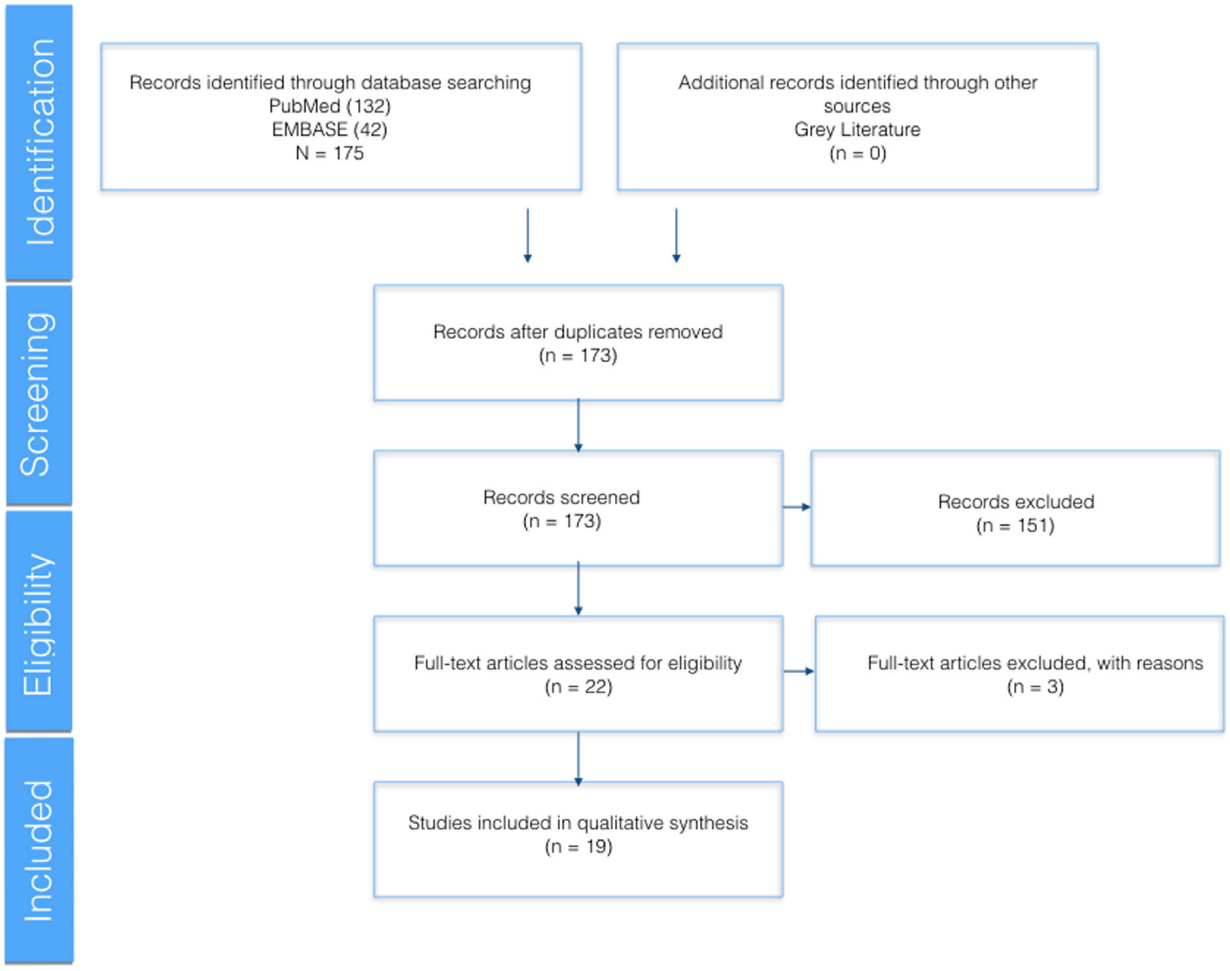
Flow chart of the selection of articles according to the PRISMA criteria (12).

The Medline (PubMed) and EMBASE search yielded 175 articles. After eliminating duplicates, a total of 173 articles were subjected to review of the title and abstract. Only 19 studies were finally included in the systematic review (Fig. 1).

The main indication for the extraction of the 3Ms was the presence of associated disease (14-17). The existing evidence for extracting or not extracting asymptomatic 3Ms without disease was found to be inconclusive (6); monitoring was thus advised (17), with due assessment of the risk-benefit ratio (18,19).

The scientific evidence therefore suggests that erupted and impacted 3Ms should be removed in the presence of painful symptoms associated to infection, dental caries or altered periodontal health of the adjacent teeth. Likewise, removal is considered to be indicated when the molar may pose problems for planned prosthodontic, orthodontic or surgical treatments.

In view of the well documented increase in morbidity associated to impacted 3Ms (non-restorable caries, fracture, infection, periodontal disease, repeated pericoronitis, cysts and tumors), extraction is considered to be indicated if associated disease is present. In contrast, prophylactic removal is not indicated in the absence of infection or other associated diseases.

-Extraction of third molars with pericoronitis

Pericoronitis is one of the associated disease conditions in which 3M extraction is considered to be indicated. It is characterized by inflammation of the mucosa surrounding the crown of the molar, with pain and sometimes erythema, edema and localized suppuration. Patients with pericoronitis can also present regional adenopathies, fever, trismus and swallowing pain, which is common in the case of inferior 3Ms and constitutes an indication for removal of the tooth.

Nevertheless, there is some controversy regarding the extraction of 3Ms with pericoronitis. Some authors advise basing the decision on the evidence afforded by the clinical guides, with the recommendation to limit intervention to the monitoring of those patients with one or two episodes of mild pericoronitis, associated to periodontal therapy and the maintenance of low bacterial plaque levels (20,21).

A study published by Tang *et al.* (22), involving 113 patients with symptoms of pericoronitis, considered that patient opinion should be taken into account in deciding treatment, since 79 patients chose extraction mainly because of altered oral function, with a minimum impact upon quality of life (odds ratio [OR] 3.22; 95% confidence interval [95%CI]: 1.08-9.58).

Apart from 3Ms in a favorable position that have caused one or two episodes of mild transient pericoronitis which can be controlled by periodontal treatment with the maintenance of a low bacterial plaque levels, the rest of 3Ms causing more severe and repeated infection should be removed (17).

Although the surgical management of pericoronitis is subject to controversy, this disorder is currently the most frequent reason for removing impacted 3Ms, particularly because patients with active pericoronal tissue infection stand to benefit from improved quality of life over the long term – this being a factor to be taken into account when considering possible extraction (21).

-Periodontal condition of the second molar

In some cases, as in the study published by Blakey *et al.* (15), the absence of symptoms of impacted 3Ms does not imply the absence of disease. In a sample of 329 patients, 25% of the second molars (2Ms) and 35% of the 3Ms presented a probing depth (PD) of > 5 mm. In this regard, the observation of signs of periodontal disease in asymptomatic 2Ms and 3Ms constitutes an unexpected finding (15).

These observations suggest that patients who wish to preserve their molars should undergo periodic clinical and radiographic evaluations in order to detect disease before it begins to cause symptoms (6,19).

On the other hand, the factors giving rise to postoperative complications comprise patient-related characteristics, anatomical factors, surgical factors and the type of associated disease (16,17). According to Chuang *et al.* (16), the degree of impaction, pre-existing infection and associated disease of 3Ms are associated to an increased risk of postoperative inflammatory complications. Among individuals with preoperative infection, 25% experienced postoperative inflammatory problems (OR 1.25; 95%CI: 1.01-1.6), while those with associated disease were three times more likely to suffer such postoperative complications (OR 3.0; 95%CI 2.2-4.3) (16).

Dicus-Brookes *et al.* (3) performed periodontal probing of 2Ms before and after the extraction of 3Ms, with the observation of significant differences: while 88% of the patients presented a PD of about 4 mm before extraction, this percentage decreased to only 46% after the operation (*p*<0.001). Furthermore, 61% presented at least one site with PD > 4 mm in other teeth located anterior to 2M before the operation, versus only 29% after surgery (*p*<0.001). The removal of 3Ms improved the periodontal condition of the 2Ms and of the teeth in a more anterior position, thanks to the decreased presence of oral pathogens (3).

Another similar study documented patients with PD ≥ 5 mm around 3M, with an attachment loss of 2 mm, while other molars presented PD < 4 mm with an attachment loss of 1 mm. The presence of 3Ms in young adults was associated with periodontal disease of other teeth. Extraction of the mandibular 3Ms improved the periodontal condition at the distal surface of 2M (19).

The above observations are consistent with those previously published by Dodson and Richardson in 2007 (23). These authors concluded that following 3M extraction, the periodontal health at the distal surface of 2M should remain constant or improve if the patient previously presented periodontal pockets or attachment loss. However, those individuals without associated disease of their 3Ms (i.e., with a healthy periodontal condition) were seen to be at greater risk of developing periodontal pockets distal to 2M after removal of 3M (23).

The prescription of chlorhexidine following the extraction of 3Ms in eruption processes with periodontal disease or other preoperative disorders was associated with a shorter time to recovery (less than two days on average) (14).

-Periodontal disease of the third molars

In the presence of periodontal disease of 3M, the clinician can either extract the molar or provide regular periodontal maintenance. It is not advisable to remove asymptomatic 3Ms without disease. However, extraction is indicated when periodontal pockets are detected, particularly if the patient exhibits deficient oral hygiene or periodontal maintenance is not feasible. All these factors should be evaluated in order to make an individualized management decision (17).

The extraction of 3M with signs and/or symptoms of periodontal disease improves periodontal health at the distal surface of 2M. However, in subjects with healthy periodontal tissue surrounding 2M, the extraction of 3M must be evaluated carefully, since probing depth and the clinical attachment level tend to worsen as a result (29).

-Postoperative morbidity following the extraction of symptomatic third molars

Bradshaw *et al.* (24) evaluated the effect of the extraction of 3M upon the quality of life (QoL) of individuals with symptoms of pericoronitis. They found the proportion of patients with severe pain to decrease from 32% before extraction to 3% after removal of the tooth. On the other hand, the proportion of patients with no pain or only very mild pain was seen to increase from 15% before extraction to 96% in the first days after removal of the tooth. The authors concluded that the extraction of 3Ms had a positive impact upon the quality of life of the patients with mild symptoms of pericoronitis.

The extraction of 3Ms with disease before surgery induces a delay in recovery after extraction, since postoperative morbidity is incremented as a result (12,13). According to Philips *et al.* (13), clinical recovery was delayed in those patients who already presented symptoms before removal and who needed to be seen at least once after extraction for the treatment of postoperative complications. This delay in recovery could be related to microbial colonization of the surgical wound (13).

Patient age and gender, and 3M position below the occlusal plane were significantly associated to prolonged recovery (13). Age and gender, and surgeon perceived difficulty of extraction were identified as statistically significant predictors of delayed healing. Women and patients who had symptomatic 3Ms before surgical extraction showed slower clinical recovery (13). 

Colorado-Bonnin *et al*. (25) evaluated the time needed to recover the quality of life of patients subjected to the surgical extraction of the 3Ms. They found men to report less pain than women, though the gender difference failed to reach statistical significance. The surgical removal of lower 3Ms was concluded to have a significant impact upon of the patient during the first three postoperative days. The quality of life of patients with symptomatic 3Ms and disease improved after surgical extraction. In the opinion of Krishnan *et al.* (26), the removal of symptomatic lower 3Ms appears to be the most logical treatment option.

Patients with impacted lower 3Ms are more susceptible to fracture of the mandibular angle as a complication of extraction (27). Older age is associated to an increased risk of mandibular fracture and of other complications secondary to systemic causes that may lead to the contraindication of extraction (28).

Postoperative morbidity associated to prolonged recovery was prospectively evaluated by Phillips *et al.* (30), with the identification of significant predictors such as age, gender, previous symptoms, and surgeon perceived difficulty of extraction (30). In addition, an impacted molar position below the occlusal plane was significantly associated to prolonged recovery, (Table 1,  Table 1 cont.,  Table 1 cont.-1).

**Table 1:**
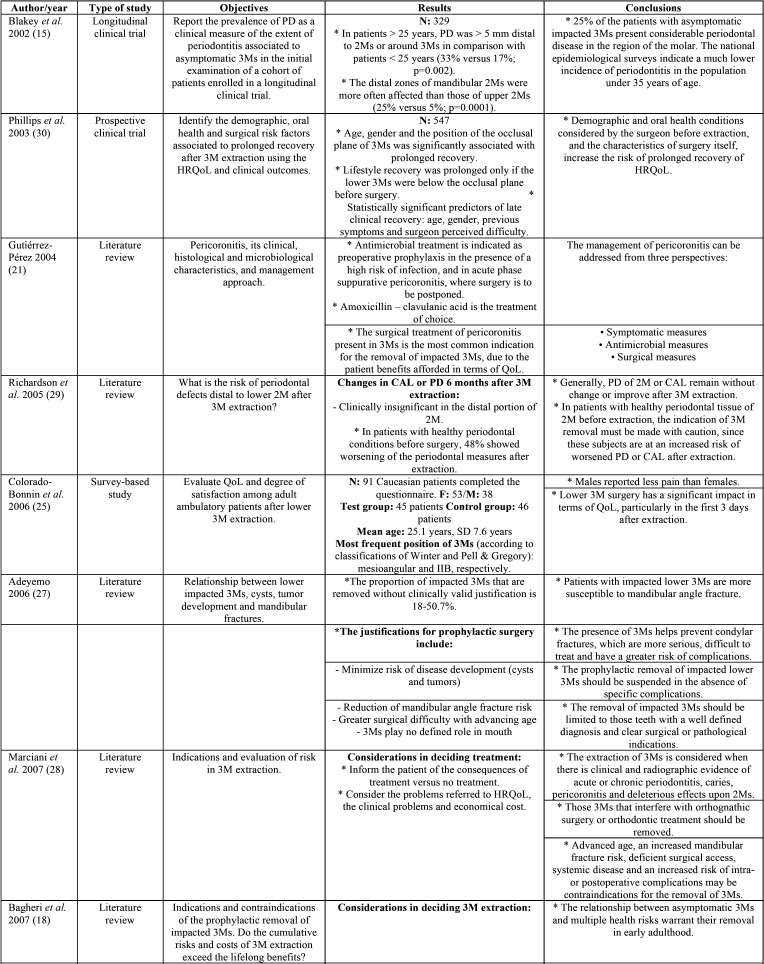
Descriptive summary of the studies included in the review.

**Table 1 cont.**
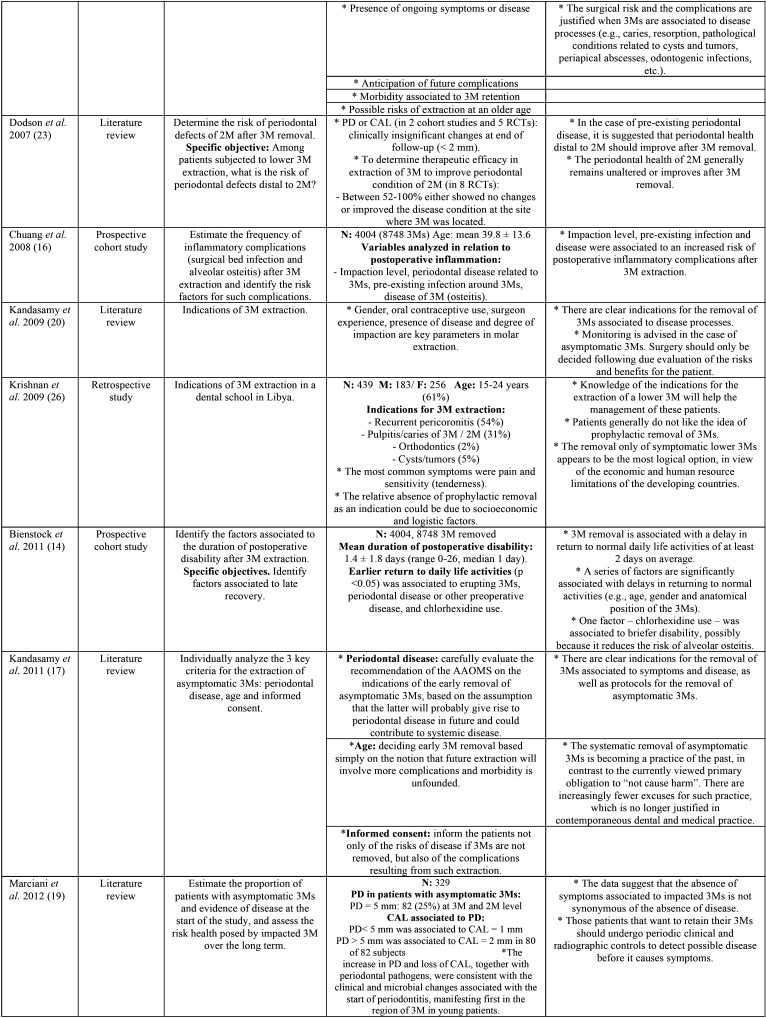
Descriptive summary of the studies included in the review.

**Table 1 cont.-1:**
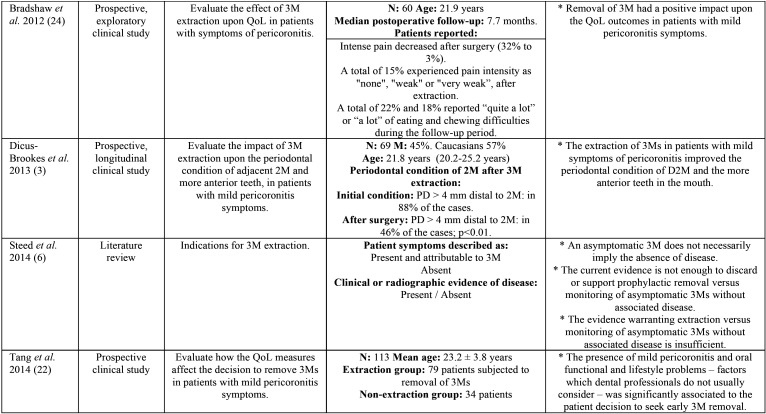
Descriptive summary of the studies included in the review.

## Discussion

The present literature review was made to determine when third molar (3M) extraction is recommended in symptomatic patients and when it is not recommended, as well as to establish the indications for the removal of impacted asymptomatic 3Ms with or without disease, and determine which cases of 3Ms with associated disease exhibit a better clinical course in terms of postoperative complications.

A literature search was made to identify those studies most relevant to the objectives of our study. The collected evidence suggests that the main indication of extraction is the presence of associated disease (14-17), though the data are not conclusive in the case of asymptomatic impacted 3Ms without associated disease (6). In such situations monitoring is advised (17), with due assessment of the risk-benefit ratio of surgical removal (18,19).

The extraction of 3Ms with pericoronitis remains subject to controversy, since the decision must be based not only on the existing evidence and surgeon experience but also on the preferences of the patient. In the case of individuals with one or two episodes of mild pericoronitis, the recommendation is to not remove the molar and to monitor the patient, ensuring good bacterial plaque control (20,21). Impaired oral functions and altered quality of life may be reasons for indicating extraction (22).

It should be noted that the absence of associated symptoms in patients with impacted 3Ms does not necessarily imply the absence of disease (15). In this regard, patients who are reluctant to accept the removal of an asymptomatic molar should undergo periodic clinical and radiological controls (6,19). Other local and demographic factors such as the level of impaction, pre-existing disease and the relationship of the molar with the occlusal plane also must be taken into account (16,17). The periodontal health of the 2M adjacent to an impacted 3M may be altered, due to the presence of periodontal pockets (3).

The evidence compiled by the present review is not intended to modify the treatment recommendations but to widen our perspective of the management of impacted 3Ms as one of the most frequent situations found in routine clinical practice. The main limitation of our study is that no recommendations were made based on the methodological quality of the studies. It was not our intention to establish such recommendations, since there were many confounding factors that precluded the drawing of firm conclusions, due to the heterogeneity of the studies included in the review.

In many cases no clear and firm evidence could be obtained; indeed, the collected data were largely imprecise – thus underscoring the need for further research in this field, on a more standardized basis and involving models of greater scientific quality.

Additional longitudinal studies are needed, exploring the evolution of periodontal disease in patients with mild pericoronitis subjected to conservative periodontal treatment without 3M extraction, compared with patients in which 3M is removed. This would help to improve our understanding of the general periodontal health impact of either 3M removal or more conservative management in the form of adequate patient monitoring. Lastly, studies are needed to analyze postoperative morbidity according to the different types of disease associated to 3M before surgical removal.

## Conclusions

Since there is a well documented increase in morbidity associated to impacted 3Ms (non-restorable caries, fracture, infection, periodontal disease, repeated pericoronitis, cysts and tumors), extraction is considered indicated in the presence of disease of the impacted molar. However, in the absence of infection or other associated disease conditions, extraction is not indicated. The extraction of 3Ms with preoperative disease results in delayed recovery after removal, since postoperative morbidity is incremented as a result.
